# Supplementary material for: Boron/Difluoroamino (B/NF2) Composites Prepared Through an Energetic Fluorinated-Centerd Surface Modification Strategy to Enhance Their Ignition and Combustion Characteristics
Source: Nanomaterials (Basel). 2024 Nov 5;14(22):1772. doi: 10.3390/nano14221772 (PMC11597125; doi:10.3390/nano14221772)
Supplement: Supplementary file 1 [file nanomaterials-14-01772-s001.zip › nanomaterials-3259832-supplementary.pdf]

nanomaterials

Supporting information

Boron/Difluoroamino (B/NF_2) Composites Prepared Through an Energetic Fluorinated-Centerd Surface Modification Strategy to Enhance their Ignition and Combustion Characteristics

Junqi He [1,2], Jing Lv [2], Yanan Li [1,2], Wenfang Zheng [1,2,*] and Renming Pan [1,2]

[1] School of Safety Science and Engineering (School of Emergency Management), Nanjing University of Science and Technology, Nanjing 210094, China; hejunqi66@sina.com (J.H.); liyanankyzy@yeah.net (Y.L.); panrenming@njust.edu.cn (R.P.)
[2] School of Chemistry and Chemical Engineering, Nanjing University of Science and Technology, Nanjing 210094, China; lvjing9487@163.com
* Correspondence: zhwf@njust.edu.cn

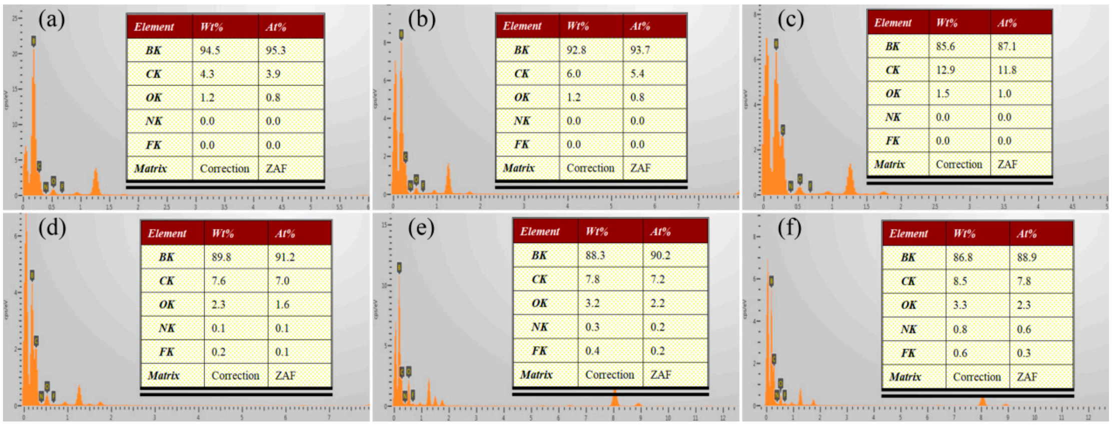

Figure S1. EDS images: **(a)** B (10–20 μm); **(b)** B (<5 μm); **(c)** B (0.5–2 μm); **(d)** B/PDB (10–20 μm); **(e)** B/PDB (<5 μm); **(f)** B/PDB (0.5–2 μm).

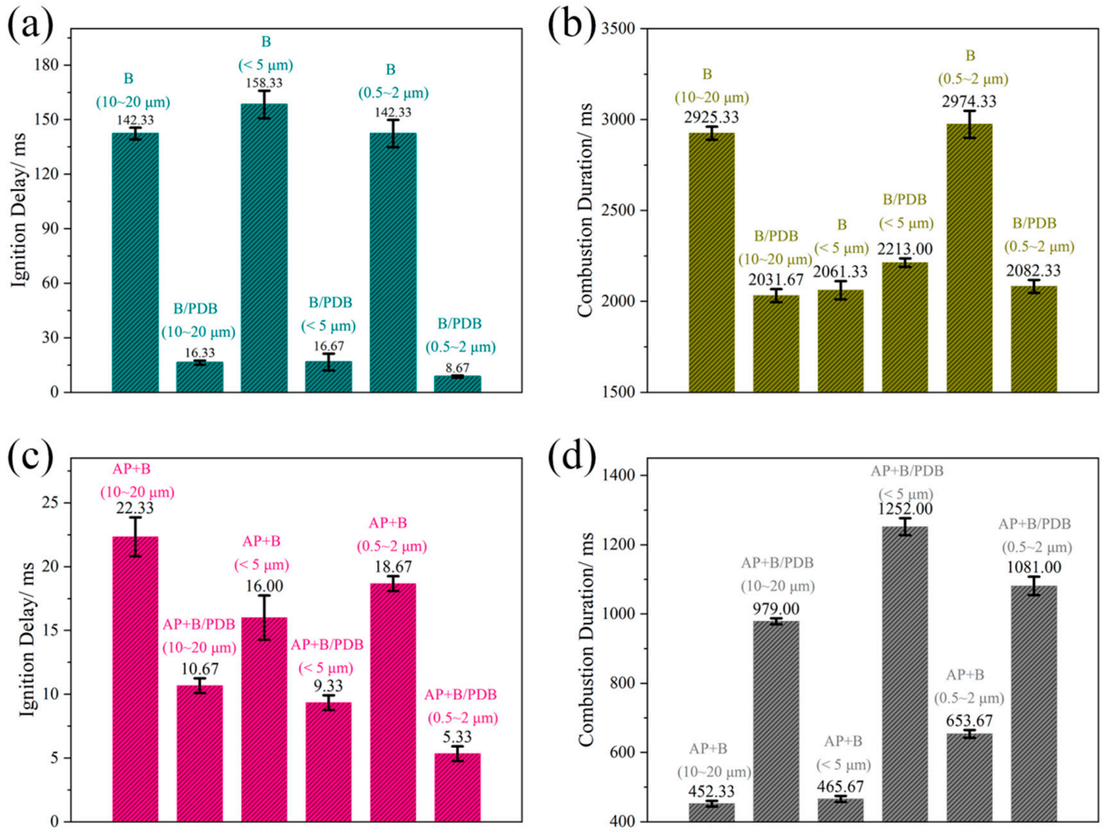

Figure S2. Statistics of ignition combustion data: (a) Ignition delay of B and B/PDB; **(b)** combustion duration of B and B/PDB; **(c)** ignition delay of mix samples(AP and B or B/PDB); **(d)** combustion duration of mix samples(AP and B or B/PDB).

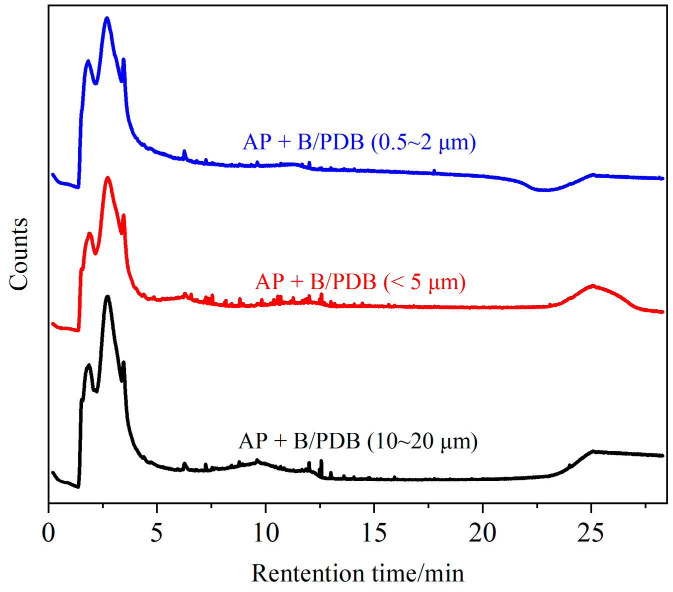

Figure S3. Chromatograms for AP and B/PDB at 300 ℃.

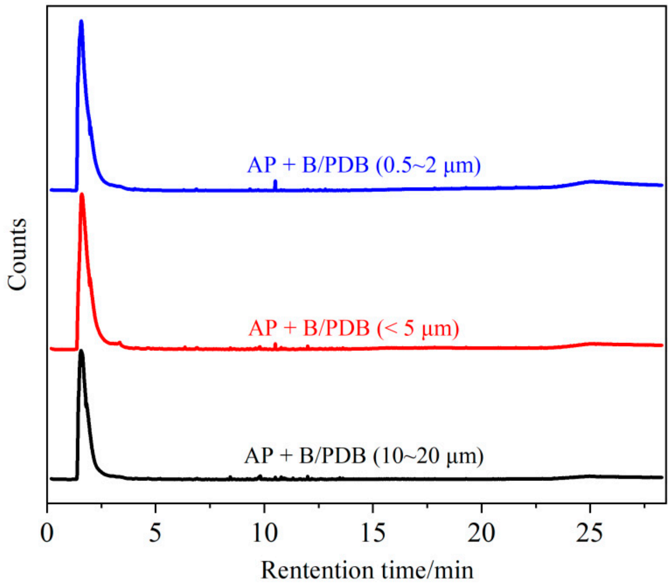

Figure S4. Chromatograms for AP and Al/PDF at 500 °C.

Table S1. The main chemical compounds of AP and B/PDB (10–20 μm) using PY/GC-MS.

Temperature/°C	Retention time/min	Compound	Chemical formula
	1.856	Hydrogen chloride	HCl
	2.149	Chlorine	Cl_2
	2.727	Ethenyl formate	$C_3H_4O_2$
	3.456	Ethylisopropylamine	$C_5H_{13}N$
	6.268	Geranyl phenylacetate	$C_{18}H_{24}O_2$
	7.246	Cyanopyrazine	$C_5H_3N_3$
	9.61	2-Ethenyl-4,5-dihydro-Oxazole	C_5H_7NO
	12.003	1,2-Benzenedicarboxylic acid	$C_8H_6O_4$
	12.487	1,3-Diisocyanato-2-methyl-Benzene	$C_9H_6N_2O_2$
500	1.569	Hydrogen chloride	HCl
	4.077	Chlorobenzene	C_6H_5Cl
	4.644	Trimethylfluorosilane	C_3H_9FSi
	5.204	1,3-Dichlorobenzene	$C_6H_4Cl_2$
	8.288	Acetophenone	C_8H_8O
	8.439	Benzoyl chloride	C_7H_5ClO
	8.589	2-Chloro-1,4-benzoquinone	$C_6H_3ClO_2$
	9.802	Benzoic acid	$C_7H_6O_2$
	10.009	1,2,3-Trichlorobenzene	$C_6H_3Cl_3$
	10.774	2,6-Dichloro-1,4-benzoquinone	$C_6H_2Cl_2O_2$
	11.994	Phthalic anhydride	$C_8H_4O_3$
	13.472	2-(3,3,3-Trifluoropropyl)piperidine	$C_8H_{14}F_3N$
	13.617	2'-[(Trimethylsilyl)oxy]propiophenone	$C_{12}H_{18}O_2Si$
	17.847	Hexamethylcyclotrisiloxane	$C_6H_{18}O_3Si_3$
	19.294	Benzoic acid,2,2-bis(trifluoromethyl) -1-aziridinyl ester	$C_{11}H_7F_6NO_2$
	20.532	4'-Methoxy-3-(3,4,5-trifluorophenyl)propiophenone	$C_{16}H_{13}F_3O_2$

Table S2. The main chemical compounds of AP and B/PDB (<5 μm) using PY/GC-MS.

Temperature/°C	Retention time/min	Compound	Chemical formula
300	1.889	Cyclobutanol	C_4H_8O
	2.727	Chlorine	Cl_2
	3.455	2-Vinyl-4,5-dihydrooxazole	C_5H_7NO
	6.283	2,3-Pyridinedicarbonitrile	$C_7H_3N_3$
	6.569	1-Octen-3-ol	$C_8H_{16}O$
	7.25	Phthalic anhydride	$C_8H_4O_3$
	7.389	3-Ethyl-3-methylhexane	C_9H_{20}
	7.545	4-Methyldecane	$C_{11}H_{24}$
	8.17	Tolylene-2,4-diisocyanate	$C_9H_6N_2O_2$
	10.558	2,3-Dimethylnonane	$C_{11}H_{24}$
	11.276	2-Methylundecane	$C_{12}H_{26}$
	12.009	Decyl isobutyrate	$C_{14}H_{28}O_2$
	12.568	Phenethyl(triethylsilyl) ether	$C_{14}H_{24}OSi$
500	1.603	Hydrogen chloride	HCl
	3.018	Chloro(chloromethoxy)methane	$C_2H_4Cl_2O$
	3.157	Methyl nitrate	CH_3NO_3
	3.336	Fluorotrinitromethane	CFN_3O_6
	4.65	Chlorobenzene	C_6H_5Cl
	6.351	Cyanopyrazine	$C_5H_3N_3$
	6.902	1,2-Dichlorobenzene	$C_6H_4Cl_2$
	7.313	Benzoic acid	$C_7H_6O_2$
	8.441	Benzoyl chloride	C_7H_5ClO
	9.22	(1-Chlorovinyl)benzene	C_8H_7Cl
	9.595	2,4-Difluorobenzonitrile	$C_7H_3F_2N$
	9.782	1,3,5-Trichlorobenzene	$C_6H_3Cl_3$
	10.506	1,2-Dichloroethylbenzene	$C_8H_8Cl_2$
	10.776	2,6-Dichloro-1,4-benzoquinone	$C_6H_2Cl_2O_2$
	12.564	3,4-Dichlorophenyl isocyanate	$C_7H_3Cl_2NO$
	13.007	Phthalic anhydride	$C_8H_4O_3$
	13.113	Tolylene-2,4-diisocyanate	$C_9H_6N_2O_2$
	13.62	2-(3,3,3-Trifluoropropyl)piperidine	$C_8H_{14}F_3N$
	15.62	Diphenyldifluorosilane	$C_{12}H_{10}F_2Si$
	17.842	Hexamethylcyclotrisiloxane	$C_6H_{18}O_3Si_3$
	19.287	Benzoic acid, 3-pentafluoropropionyloxy-,trimethylsilyl ester	$C_{13}H_{13}F_5O_4Si$
	21.585	*N*-[(Pentafluorophenyl)methylene]-3,4-bis[(trimethylsilyl)oxy]benzeneethanamine	$C_{21}H_{26}F_5NO_2Si_2$

Table S3. The main chemical compounds of AP and B/PDB (0.5–2 μm) using PY/GC-MS.

Temperature/°C	Retention time/min	Compound	Chemical formula
300	1.826	Vinyl formate	$C_3H_4O_2$
	2.686	Chlorine	Cl_2
	3.459	Cyanopyrazine	$C_5H_3N_3$
	6.262	Norvaline	$C_5H_{11}NO_2$
	12.049	Vinyl benzoate	$C_9H_8O_2$
	14.156	*N*-Methyl nicotinimidate, *O*-trimethylsilyl	$C_{10}H_{16}N_2OSi$
	17,761	Benzoic acid,2,2-bis(trifluoromethyl) -1-aziridinyl ester	$C_{11}H_7F_6NO_2$
500	1.574	Hydrogen chloride	HCl
	4.046	Benzoic acid	$C_7H_6O_2$
	6.299	1,2-Dichlorobenzene	$C_6H_4Cl_2$
	6.868	1,2,3-Trichlorobenzene	$C_6H_3Cl_3$
	7.307	1-(5-Fluoro-2-hydroxyphenyl)-1-ethanone	$C_8H_7FO_2$
	8.437	2,6-Dichloro-1,4-benzoquinone	$C_6H_2Cl_2O_2$
	9.344	4-Cyclopentene-1,3-dione,2,2,4,5-tetrachloro	$C_5Cl_4O_2$
	9.714	1,2-Benzenedicarboxylic acid	$C_8H_6O_4$
	10.083	3-Nitro-1-phenylpropan-1-one	$C_9H_9NO_3$
	10.504	Hexamethylcyclotrisiloxane	$C_6H_{18}O_3Si_3$
	10.776	Tolylene-2,4-diisocyanate	$C_9H_6N_2O_2$
	10.915	4-Chlorophthalic acid	$C_8H_5ClO_4$
	11.812	Octamethylcyclotetrasiloxane	$C_8H_{24}O_4Si_4$
	12	4-Chloro-1-methyl-1,2-dihydro-1,5-naphthyridin-2-one	$C_9H_7ClN_2O$
	12.565	Propyphenazone	$C_{14}H_{18}N_2O$
	12.809	Benzoic acid, 4-trifluoroacetyloxy-, trimethylsilyl ester	$C_{12}H_{13}F_3O_4Si$
	13.362	1,1,3,3,5,5,7,7-Octamethyltetrasiloxane	$C_8H_{26}O_3Si_4$
	13.621	Hexasiloxane,1,1,3,3,5,5,7,7,9,9,11,11-dodecamethyl	$C_{12}H_{38}O_5Si_6$
	14.646	Dodecamethylcyclohexasiloxane	$C_{12}H_{36}O_6Si_6$
	20.778	*N*-(Trifluoroacetyl)-*N,O,O',O''*-tetrakis(trimethylsilyl)norepinephrine	$C_{22}H_{42}F_3NO_4Si_4$
	22.666	*N*-[(Pentafluorophenyl)methylene]-3,4-bis[(trimethylsilyl)oxy]benzeneethanamine	$C_{21}H_{26}F_5NO_2Si_2$

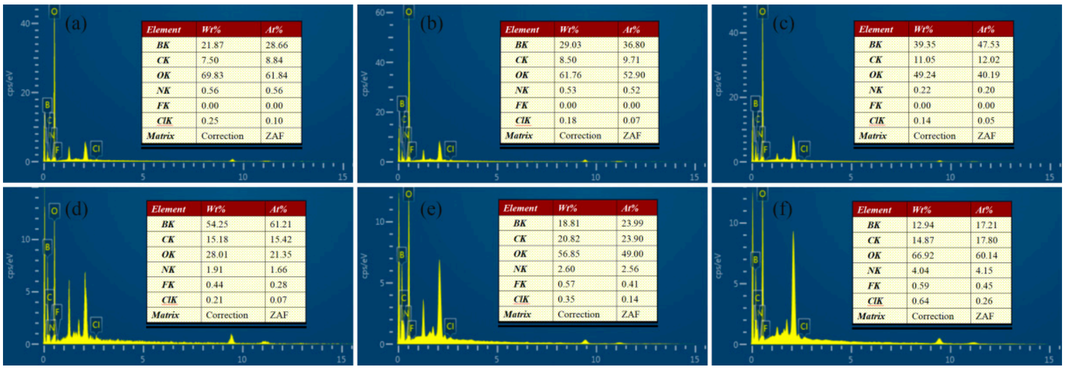

Figure S5. EDS for collected combustion residues of mixtures of AP : **(a)** B (10–20 μm); **(b)** B (<5 μm); **(c)** B (0.5–2 μm); **(d)** B/PDB (10–20 μm); **(e)** B/PDB (<5 μm); **(f)** B/PDB (0.5–2 μm).
